# Can phylogeny predict chemical diversity and potential medicinal activity of plants? A case study of amaryllidaceae

**DOI:** 10.1186/1471-2148-12-182

**Published:** 2012-09-14

**Authors:** Nina Rønsted, Matthew R E Symonds, Trine Birkholm, Søren Brøgger Christensen, Alan W Meerow, Marianne Molander, Per Mølgaard, Gitte Petersen, Nina Rasmussen, Johannes van Staden, Gary I Stafford, Anna K Jäger

**Affiliations:** 1 Botanic Garden, Natural History Museum of Denmark, Sølvgade 83, Opg. S, Copenhagen, DK-1307, Denmark; 2 Natural Products Research, Drug Design and Pharmacology, Faculty of Health and Medical Sciences, University of Copenhagen, Universitetsparken 2, Copenhagen, DK-2100, Denmark; 3 Centre for Integrative Ecology, School of Life and Environmental Sciences, Deakin University, 221 Burwood Highway, Burwood, Victoria, 3125, Australia; 4 USDA-ARS-SHRS, National Germplasm Repository, 13601 Old Cutler Road, Miami, Florida, USA; 5 Fairchild Tropical Garden, Miami, Florida, USA; 6 Research Centre for Plant Growth and Development, School of Life Sciences, University of KwaZulu-Natal Pietermaritzburg, Private Bag X01, Scottsville, 3201, South Africa

**Keywords:** Amaryllidaceae, Phylogeny, Chemical diversity, Prediction, Lead discovery

## Abstract

**Background:**

During evolution, plants and other organisms have developed a diversity of chemical defences, leading to the evolution of various groups of specialized metabolites selected for their endogenous biological function. A correlation between phylogeny and biosynthetic pathways could offer a predictive approach enabling more efficient selection of plants for the development of traditional medicine and lead discovery. However, this relationship has rarely been rigorously tested and the potential predictive power is consequently unknown.

**Results:**

We produced a phylogenetic hypothesis for the medicinally important plant subfamily Amaryllidoideae (Amaryllidaceae) based on parsimony and Bayesian analysis of nuclear, plastid, and mitochondrial DNA sequences of over 100 species. We tested if alkaloid diversity and activity in bioassays related to the central nervous system are significantly correlated with phylogeny and found evidence for a significant phylogenetic signal in these traits, although the effect is not strong.

**Conclusions:**

Several genera are non-monophyletic emphasizing the importance of using phylogeny for interpretation of character distribution. Alkaloid diversity and *in vitro* inhibition of acetylcholinesterase (AChE) and binding to the serotonin reuptake transporter (SERT) are significantly correlated with phylogeny. This has implications for the use of phylogenies to interpret chemical evolution and biosynthetic pathways, to select candidate taxa for lead discovery, and to make recommendations for policies regarding traditional use and conservation priorities.

## Background

During evolution, plants and other organisms have developed a diversity of chemical defence lines, leading to the evolution of various groups of specialized metabolites such as alkaloids, terpenoids, and phenolics, selected for their endogenous biological function [1-7]. Intuitively, a correlation between phylogeny and biosynthetic pathways is sometimes assumed [1,8-10] and could offer a predictive approach enabling deduction of biosynthetic pathways [6,11-15], defence against herbivores [16,17], more efficient selection of plants for the development of traditional medicine and lead discovery [18-22] as well as inform conservation priorities [23].

Several studies have confirmed the usefulness of specialized metabolites such as glucosinolates, iridoids, sesquiterpene lactones, flavonoids, and phenolics to support molecular based phylogenies contradicting morphologic patterns [11,12,24-29]. On the contrary, several studies have found inconsistency of specialized metabolite profiles at various taxonomic levels and indicated that specialized chemistry and anti-herbivore defence syndromes tend to be poorly correlated with plant phylogeny [6,7,13,30].

Lack of congruence between specialized chemistry and phylogeny may be caused by several different phenomena. One contributing factor is convergent evolution by which the same or similar traits originate independently in taxa that are not necessarily closely related, often in response to similar environmental challenges [17,31]. A striking example of convergent evolution is the common use of the sex pheromone (Z)-7-dodecen-l-yl acetate by over 120 species of primarily Lepidopteran insects and female Asian elephants, *Elephas maximus*[32]. In relation to plants, well known convergent morphological adaptations are the occurrence of prickles, thorns, and spines, which have evolved to avoid or limit herbivory [33], succulence as adaptation to dry environments in both North American Cactaceae and African *Euphorbia*[34,35], and insectivorous plants, which have evolved several times in response to a nitrogen-deficient environment [36]. Likewise, chemical defence lines may also have arisen independently in unrelated taxa, and convergent evolution in plant specialized metabolism appears to be surprisingly common [6,17,31,37]. For example, the ability to produce cyanogenic glycosides appears to have evolved independently in many different plant families [17,31].

However, convergent evolution can be difficult to verify because absence of evidence is not evidence of absence and it is possible that some compounds presently considered to be limited to some lineages are indeed more universally found in plants [31]. Specialized compounds are not continuously expressed, but may be produced as a response to herbivory or other damage, the expression may also be dependent on the environment [38] and plants often use a combination of several defensive traits [7,17]. In addition, chemosystematic data are scattered in the literature and negative results are often not reported. Absence or presence of a compound is also dependent on the amount of plant material investigated as well as the detection limit of the analytical methods [27,39]. Finally, the existence of several different phytochemical methods can cause inconsistence in the results reported in the literature.

Nevertheless, reports of incongruence between phytochemistry and phylogeny have questioned the degree of correlation between phylogeny and specialized metabolites, indicating that such a correlation cannot simply be assumed [6]. However, this relationship has rarely been tested because of lack of accurate estimates of phylogeny and corresponding chemical data; lack of tradition for interdisciplinary studies bridging botany, chemistry, and molecular systematics; and appropriate statistical tools. Consequently, the potential predictive power is unknown [17]. In the present study, we use Amaryllidaceae subfamily Amaryllidoideae as a model system for testing the correlation between phylogenetic and chemical diversity and biological activity.

**Figure 1:**
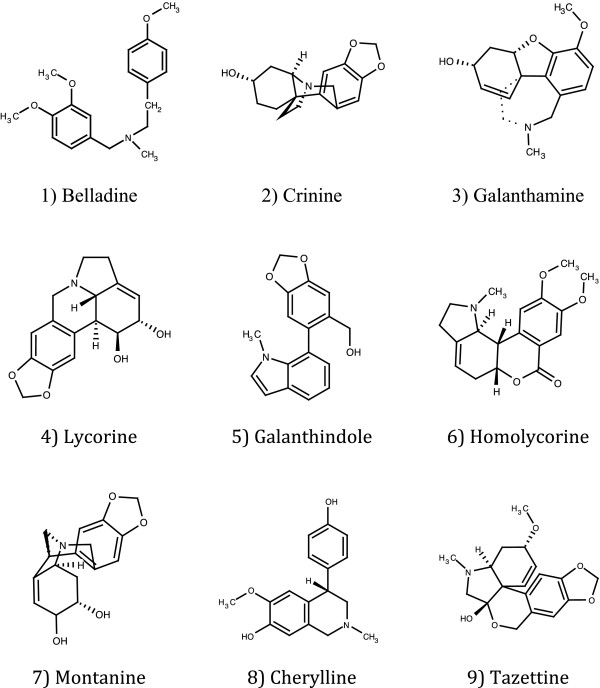
**Alkaloid types recovered in the present study.** Alkaloids were classified to type according to the hypothetical biosynthetic pathways proposed by Jin [45,46]. Marvin was used for drawing and displaying chemical structures, Marvin 5.9.0, 2012, ChemAxon (http://www.chemaxon.com).

Amaryllidaceae subfamily Amaryllidoideae sensu APG III [40] (formerly recognized as a separate family, Amaryllidaceae J.St.-Hil.) is a widely distributed subfamily of 59 genera and about 850 species. Amaryllidoideae has centres of diversity in South Africa, South America, particularly in the Andean region, and in the Mediterranean, three of the recognized hotspots of biodiversity on Earth [41,42] (http://www.biodiversityhotspots.org). Plants of the Amaryllidoideae are used in traditional medicine to treat mental problems, primarily in Southern Africa [43,44]. The traditional use of plants of Amaryllidoideae has been related to their unique and subfamily specific alkaloid chemistry (Figure 1). Over 500 alkaloids have been described from various species and have been subdivided into 18 major types based on hypothetical biosynthetic pathways [45-47]. Extracts or isolated alkaloids of Amaryllidaceae species have shown activity *in vitro* in a range of assays related to disorders of the central nervous system, primarily Alzheimers disease (inhibition of acetylcholinesterase, AChE) [21,48-51], and anxiety and depression (affinity to the serotonin re-uptake transporter, SERT) [21,52,53]. Galanthamine is registered in a number of countries as an AChE inhibitor (Reminyl or Rezadyne; Janssen Pharmaceutica) [54]. Another Amaryllidaceous alkaloid, sanguinine (9-O-demethylgalanthamine), is shown to be a ten times more potent inhibitor of AChE than galanthamine *in vitro*[55].

Amaryllidaceae subfamily Amaryllidoideae is therefore an ideal model system for comparing phylogenetic and chemical diversity with bioactivity. Previous molecular phylogenetic studies based on plastid gene regions (*rbcL, trnLF,* and *ndhF*) have confirmed Amaryllidoideae as monophyletic and resolved many taxa into geographically confined monophyletic groups [42,56]. The African tribe Amaryllideae has been well supported as sister group to the remaining taxa. However, the relationship among several other early diverging lineages, in particular the African tribes Haemantheae and Cyrtantheae, and the Australasian Calostemmateae are not well supported by previous studies and remain problematic [42]. In a study by Meerow and Snijman [42] based on parsimony analysis of plastid *ndhF* sequences, Amaryllideae also resolved as sister to the remainder of the subfamily. The next major split resolved a clade with American and Eurasian subclades, and an African/Australasian clade with Cyrtantheae as sister to a Haemantheae/Calostemmateae clade. However, this African/Australasian clade was not supported by bootstrap analysis.

The objectives of the present study were: (1) to produce a comprehensive and well supported phylogenetic hypothesis of Amaryllidaceae subfamily Amaryllidoideae based on total evidence from DNA regions from all three plant genomes; (2) to test for correlation between phylogenetic and chemical diversity and central nervous system (CNS) related activities.

## Results

### Phylogeny

**Figure 2:**
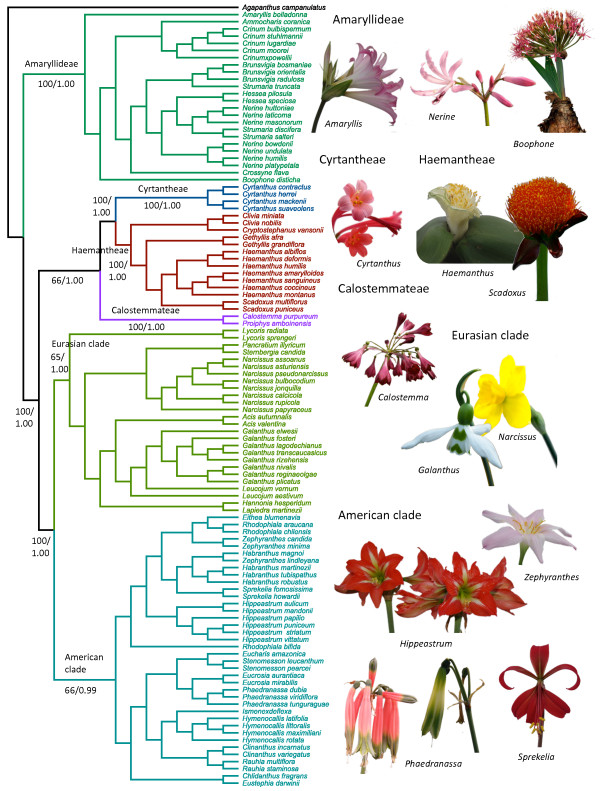
**Phylogenetic hypothesis for Amaryllidaceae subfamily Amaryllidoideae.** Obtained after 1,000,000 replicates of Bayesian inference. Parsimony bootstrap percentages and Bayesian posterior probabilities (BS/PP) are indicated for major clades only. Examples of members are illustrated on the right hand side.

The ITS region was the most informative region followed by the *matK* region (Table 1). The *trnLF* and the *nad1* regions resolved 10% or less of the clades with strong support defined as ≥ 90% bootstrap support. There were no strongly supported conflicts among any regions (Bayesian consensus tree with posterior probabilities and parsimony bootstrap consensus tree for the total evidence analysis and bootstrap consensus trees of individual regions are provided as Additional file 1: Figures S1-S7). The number of resolved clades (87%; Table 1) and the number of resolved clades supported by **≥** 90% bootstrap (62%) was highest in the total evidence analysis, which was also the only analysis that resolved all the major lineages. The Bayesian analysis of the total evidence matrix provided the same overall topology as parsimony analysis and all major clades were strongly supported by Bayesian analysis (Figure 2; Additional file 1: Figures S1 and S2).

**Table 1:** Details of the matrices included in this study

**Matrix**	**# taxa**	**# aligned characters**	**# of PPI**^**1**^**(%)**	**# MP trees**	**Length of MP trees**	**CI**^2^	**RI**^3^	**Percent clades with > 50% BS**^**4**^	**Percent clades with ≥ 90% BS**^**4**^
**ITS**	105	953	502 (53)	6520	2537	0.42	0.82	78	55
***trnLF***	106	1163	185 (16)	470	601	0.70	0.86	33	10
***matK***	105	2019	295 (15)	9940	922	0.74	0.90	67	39
**Plastid combined**	107	3182	480 (15)	3620	1544	0.71	0.88	76	45
***nad1***	104	1726	53 (3)	8330	275	0.79	0.97	28	7
**Total evidence**	109	5861	1086 (19)	554	4454	0.53	0.85	87	62

^1^PPI: potentially parsimony informative characters. ^2^CI: Consistency index. ^3^RI: Retention index. ^4^Percent of resolved clades in the Bootstrap consensus tree with > 50% BS (bootstrap support) and with **≥** 90% BS are proportions of the possible number of clades (# taxa −1).

The topology (Figure 2) of the total evidence analysis largely supports previous studies [42,56]. The African tribe Amaryllideae (100% BS; PP = 1.00) is sister to the remainder of the Amaryllidoideae (100% BS; PP = 1.00), and this is strongly supported by all analyses. The next major split resolves an American clade (66% BS; PP = 0.99) and a Eurasian clade (65% BS; PP = 1.00) as sisters (100% BS; PP = 1.00) and a clade (66% BS; PP = 1.00) with the African monogeneric tribe Cyrtantheae (100% BS; PP = 1.00), and tribe Haemantheae (100% BS; PP = 1.00) as sisters (100% BS; PP = 1.00), and the Australasian tribe Calostemmateae (100% BS; PP = 1.00) as sister to these.

In the ITS analysis ( Additional file 1: Figure S3), tribe Amaryllideae (100% BS) is sister to the remainder of Amaryllidoideae (61% BS). Within the remainder of Amaryllidoideae, tribe Calostemmateae (100% BS) is sister to a clade (54% BS) including tribes Cyrtantheae (100% BS), Haemantheae 95% BS) and the American and European Amaryllidoideae. Tribes Cyrtantheae and Haemantheae are sisters (96% BS). In the combined plastid analysis ( Additional file 1: Figure S6), Cyrtantheae is sister to the remainder of Amaryllidoideae except tribe Amaryllideae (75% BS). In both the *matK* ( Additional file 1: Figure S4 supporting online material) and the combined plastid analysis tribe Calostemmateae is sister to tribe Haemantheae (*matK*: 70% BS; plastid: 94% BS).

The low bootstrap support (65%) for the Eurasian clade in the total evidence analysis (Figure 2, Additional file 1: Figure S2) may be caused by uncertainty in the placement of the genus *Lycoris*. The remainder of the Eurasian clade is strongly supported in all analyses except *trnLF* and *nad1*, which are the two regions providing the least resolution and support in general ( Additional file 1: Figures S3-S7 in the supporting online material).

### Relationship of phylogeny to chemistry and bioactivity

The relationship of individual types of compounds and biological activity could be assessed using the *D* metric developed to deal with discrete binary characters [57]. *D* is equal to 1 if the observed chemical component has a random distribution (i.e. no phylogenetic signal). *D* is equal to 0 if the component is distributed exactly as would be predicted under a Brownian motion model of gradual divergent evolution (i.e. strong phylogenetic signal). See Materials and Methods for details. Of the seven alkaloid types which are found in more than one species we found evidence for some phylogenetic signal of five types (Table 2a) [57]. With the exception of tazettine and galanthindole, *D* values were significantly different from that expected under a random distribution of the components across the phylogenetic tree (*D* = 1). Inhibition of acetylcholinesterase (AChE) and binding to the serotonin re-uptake transporter (SERT) were used as proxies for biological activity. Both measures of bioactivity, AChE activity and SERT activity also displayed significant phylogenetic signal (Table 2b). However, in none of the above cases was the phylogenetic signal sufficiently strong to be considered as indistinguishable from a Brownian motion model of evolution, where traits are strongly ‘clumped’ on the phylogeny (and *D* = 0) [57].

**Table 2:** **Phylogenetic signal in chemistry and biological activity determined using Fritz and Purvis’s**[57]***D*metric (see Materials and Methods for details)**

			
**a) Chemical components**			
**Alkaloid group**	***D***	**P (D = 1)**	**P (D = 0)**
Crinine	0.6768	0.021	0
Galanthamine	0.549	0	0.009
Lycorine	0.77	0.018	0
Galanthindole	1.091	0.621	0.011
Homolycorine	0.769	0.021	0
Montanine	0.572	0.002	0.01
Tazettine	0.852	0.094	0
**b) Biological activity**			
**Measure**	***D***	**P (D = 1)**	**P (D = 0)**
AChE	0.679	0.004	0.001
SERT	0.634	0.037	0.044

*D* is equal to 1 if the observed chemical component has a random distribution (i.e. no phylogenetic signal). *D* is equal to 0 if the component is distributed exactly as would be predicted under a Brownian motion model of gradual divergent evolution (i.e. strong phylogenetic signal). P values represent the probability that the observed *D* value is equal to 1 or 0, respectively (P >0.05 indicates that the observed value is not significantly different from these values).

There was a statistically highly significant correlation between differences in chemical profile and phylogenetic distance, indicating that closely-related species tend to have more similar chemical profiles than more distantly-related species, although the effect was not strong (Mantel test: r = 0.085, p = 0.002). There was also statistically significant correlation between chemical profile and phylogenetic distance in the genus level comparison (Mantel test: r = 0.090, p = 0.024), although the effect was also weak.

## Discussion

### Phylogeny of Amaryllidoideae

For the purpose of the present study, we consider the total evidence approach to provide the best estimate of phylogeny and all major lineages are supported by both parsimony and Bayesian analyses. The present study has doubled previous sampling of Amaryllidoideae from 51 species [56] to 108 and from combined analysis of two DNA regions [42,56] to four DNA regions representing all three genomes. The only previous study resolving relationships among basal lineages was based on plastid *ndhF* sequences [42] and resolved Calostemmateae and Haemantheae as sisters and tribe Cyrtantheae as sister to these. However, in the present study (Figure 2), the African tribes Cyrtantheae and Haemantheae are strongly supported by both bootstrap and Bayesian posterior probabilities as sister clades. Tribe Calostemmateae is sister to these, although this was only weakly supported by the bootstrap, but strongly supported by Bayesian posterior probabilities. A sister group relationship of the two African tribes Cyrtantheae and Haemantheae and the Australasian tribe Calostemmateae as sister to these appears more convincing than the alternative based on biogeography. However, in terms of morphology there may be some room to question this relationship. The indehiscent capsule of Calostemmateae has more in common with the indehiscent baccate fruit of Haemantheae (resembling the unripe fruit of *Clivia*, *Scadoxus*, *Haemanthus,* and *Cryptostephanus*) than with the dehiscent capsule of *Cyrtanthus*.

### Phylogenetic signal of chemical diversity and bioactivity

Our approach to quantify overall correlation between chemical and phylogenetic diversity has previously been applied to show positive correlations between pheromone differences and nucleotide divergence in *Bactrocera* fruit flies [58] and phylogenetic correlation of cuticular hydrocarbon diversity in ants [59]. We have now shown the potential application of this approach to explore correlations between phylogenetic and chemical diversity of medicinal plants.

**Figure 3:**
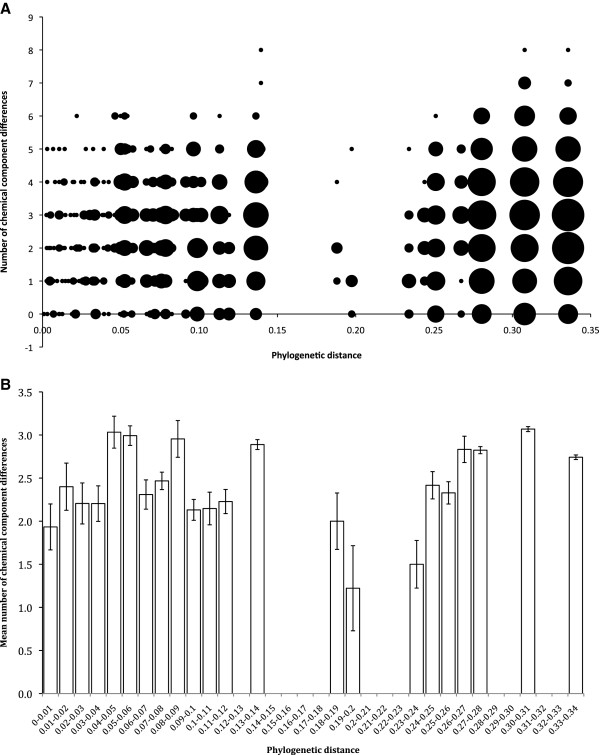
**Relationship between phylogenetic distance and chemical diversity (number of alkaloid group differences) for Amaryllidaceae subfamily Amaryllidoidae. A**) Scatter plot showing distribution of points and best-fit regression line. The size of each point is proportional to the log-transformed number of data points contributing to it. **B**) The same data showing mean (±s.e.) number of differences for categories of phylogenetic distance (number of substitutions per site) for ease of visualization. The overall relationship is significant. There is a statistically highly significant correlation between differences in chemical profile and phylogenetic distance, although the effect is not strong (Mantel test: r = 0.085, p = 0.002), indicating that closely related species tend to have more similar chemical profiles than more distantly related species.

We found significant phylogenetic signal for five out of seven tested individual alkaloid types and for both AChE and SERT bioactivity proxies, although the signal was not strong enough to be indistinguishable from a Brownian model of evolution, where traits are strongly clumped on the phylogeny [57]. There was also a highly significant correlation between differences in chemical profiles and phylogenetic distance in both species and generic level analyses. Despite this, there was often still considerable difference in chemical make-up even between phylogenetically very close species (Figure 3) with the average number of alkaloid types differing between congeners being 2.26. Whereas members of some genera such as *Crinum* and *Galanthus* have generally similar chemical profiles, other genera such as *Hippeastrum* and *Narcissus* have striking diversity.

An explanation for the moderate correlation found could be either methodological artefacts or underlying ecological or genetic differences [38]. We minimized methodological artefacts by using the same plant accessions for both phylogenetic, chemical, and bioactivity studies, and by analysing our data with consistent methods. Chemical profiles were based on types deducted from hypothetical pathways and could be an oversimplification of the chemical diversity contained by over 500 individual alkaloid structures known from the subfamily [45,47].

The strength of correlation could be dependent on taxonomic scale. Whereas alkaloids derived from norbelladine and its derivatives are almost exclusively restricted to the subfamily Amaryllidoideae [45], and alkaloids with AChE activity appear to be phylogenetically constrained within *Narcissus *[18], the considerable variation at the species and genus level found in this study corresponds well with within species variation of alkaloid profiles in for example *Galanthus *[51,60].

Evaluation of extensive historical drug data, marine natural products, medicinal plants and bioactive natural products suggests that drugs are derived mostly from pre-existing drug-productive families that tend to be clustered rather than randomly scattered in the phylogenetic tree of life [61]. Zhu *et al. *[61] further suggest that efforts to identify new potential drugs can therefore be concentrated on exploring a number of drug-productive clusters. However, based on our results, such a strong presumed correlation between phylogeny and bioactivity appears to be an oversimplification, at least at the taxonomic scale tested in our study. Based on our data for the medicinally important plant subfamily Amaryllidoideae, it appears that phylogeny can predict chemical diversity and bioactivity, but considerable caution must be emphasized. We also suggest that phylogenetic correlation of chemical traits of interest may need to be assessed for a particular phylogenetic framework before it is used for prediction of occurrence in un-investigated taxa.

### Application of phylogenetic prediction and *in silico* data mining

A predictive approach could enable deduction of biosynthetic pathways, defence against herbivores, more efficient selection of plants for the development of traditional medicine and lead discovery as well as inform conservation priorities as outlined in the introduction. A plethora of data on phylogenetic relationships, chemical constituents and bioactivity are available through public databases (e.g., GenBank) and in the literature. Systematic *in silico* data mining could enable more efficient use of predictive approaches to speed up all of the above applications [22,61-65].

However, a methodological framework still needs to be developed. In the present study, we have suggested an approach for testing correlations between phylogenetic and chemical diversity and biological activity using experimental data generated for this purpose. One method for subsequent identification of specific nodes in phylogenies with high bio-screening potential has been proposed by Saslis-Lagoudakis *et al.*[22] using tools from community ecology. In this approach, a matrix of ethnomedicinal use was composed and used to identify nodes in a phylogeny of *Pterocarpus* (Fabaceae), which have more medicinal taxa related to a specific category of use than expected by chance. This approach could be useful for identifying alternative resources or substitute taxa in cases where supply of a medicinal plant or natural product of interest is limited or where species in use are subject to conservation concerns [22]. However, for the purpose of increasing the chance of making truly new discoveries such as new compounds and/or new activity profiles, it may be more relevant to identify clades that possess activity of interest and at the same time do not correspond to well known compounds with well known activity profiles [66].

Other methods for predictive *in silico* data mining may be combined with a phylogenetic selection approach, e.g., exploration of natural product chemical space as developed by Backlund and co-workers [65,67,68]. Another computerized geospatial tracking tool linking bioactive and phylogenetic diversity has been developed for microorganisms [63]. The concept of virtual parallel screening developed for natural products by Rollinger [64], which simultaneously enables fast identification of potential targets, insight into a putative molecular mechanism and estimation of a bioactivity profile, could allow for optimal selection of relevant targets.

## Conclusion

In conclusion, we have shown significant correlation between phylogenetic and chemical diversity and biological activity in the medicinally important plant subfamily Amaryllidoideae. However, a correlation cannot be assumed for other study systems without considerable caution or testing. This has implications for the use of phylogenies to interpret chemical evolution and biosynthetic pathways, to select candidate taxa for lead discovery, and to make recommendations for policies regarding traditional use and conservation priorities. Phylogenetic prediction of chemical diversity and biological activity may provide an *evolutionary* based tool alone or in combination with other recently developed tools for *in silico* data mining of natural products and their bioactivity.

## Methods

### Taxon sampling

Specimens were collected in their natural habitat or obtained from botanical gardens or specialist nurseries. Sampling included 108 (over 10%) of circa 850 species in Amaryllidaceae subfamily Amaryllidoideae [69] with *Agapanthus campanulatus* L. used as outgroup ( Additional file 2). Sampling represents 43 of circa 60 genera and all currently recognized tribes except Griffineae Ravenna [41,42,56]. Samples from tribes Galantheae and Haemantheae were partly retrieved from previous studies [21,51]. The same accessions of plant material were used for both molecular, chemical, and bioactivity analysis to minimize effects of intraspecific and ecological variation.

### Phylogenetic analyses

DNA was extracted using the Qiagen DNeasy kit (Qiagen, Copenhagen, Denmark) from 20 mg of dried leaf fragments. Amplification and sequencing of the nuclear encoded ITS and plastid encoded *matK* and *trnLF* regions followed Larsen *et al.*[51]. Amplification and sequencing of the mitochondrial *nad1* region followed Cuenca *et al.*[70]. Primers used for amplification and sequencing are listed in Additional file 3. Both strands were sequenced for each region for all taxa whenever possible. Sequences were edited and assembled using Sequencer 4.8^TM^ software (Gene Codes, Ann Arbor, MI, USA). All sequences are deposited in GenBank and accession numbers JX464256- JX464610 are listed in Additional file 2. Sequences were aligned using default options in MUSCLE [71] as implemented in the software SeaView [72].

Phylogenetic analyses were conducted using both parsimony and Bayesian inference. Most parsimonious trees (MP) were obtained with PAUP v. 4.0b10 [73] using 1,000 replicates of random taxon addition sequence and TBR branch swapping saving multiple trees. All characters were included in the analyses and gaps were treated as missing data. We analysed the four regions separately to identify strongly supported phylogenetic conflicts among the regions, prior to performing a combined total evidence analysis. By using total evidence the explanatory and descriptive power of the data is maximized [74]. Bootstrap analyses [75] of the four individual datasets and the combined dataset were carried out using 1,000 replicates. Bayesian analysis of the combined dataset was performed with MrBayes 3.1.2 [76]. We first selected the best fitting model (GTR + I + G; Parameters: lset NST = 6 RATES = gamma) of molecular evolution using the Akaike criterion (AIC) in Modeltest v. 3.8 [77]. The analysis was performed with 1,000,000 generations on four Monte Carlo Markov chains. The average standard deviation of the split frequencies was 0.01 after 232,000 generations and < 0.005 after 1 million generations corresponding to an effective sample size of 115 using the software Tracer v. 1.5.0 [78]. The first 2,500 (25%) trees of low posterior probability were deleted and all remaining trees were imported into PAUP. A majority rule consensus tree was produced showing the posterior probabilities (PP) of all observed bi-partitions. We also performed a partitioned analysis allowing different models for the three genomes. However, in consideration of the limited information present in our plastid and mitochondrial datasets (Table 1), partition rich strategies are not always the best ones and in some cases less complex strategies have performed better [79,80]. Although the Bayesian MCMC approach is good at handling complex models, there is a risk of over-parameterization, which can result in problems with convergence and excessive variance in parameter estimates [81].

### Chemical diversity

Alkaloids were extracted from 300 mg dried bulb scales using 0.1% H_2_SO_4_ and clean-up on ion-exchange solid phase columns as described by Larsen *et al.*[51]. All extracts were concentrated under vacuum until dryness and re-dissolved to a standard concentration of 5 mg ml^-1^ in MeOH. Alkaloid profiles were obtained by gas chromatography–mass spectrometry (GC-MS) as described by Larsen *et al.*[51] using a method developed by Berkov *et al.*[82]. Alkaloids were identified to type by comparison with the NIST 08 Mass Spectral Search Program, version 2.0 (NIST, Gaithersburg, Maryland) and with published spectral data. Alkaloid structures were scored to one of eighteen types (Figure 1) proposed by Jin [45,46] based on hypothetical biosynthetic pathways [51]. Only nine of the eighteen alkaloid types were recorded in the present study (Figure 1). Each type show characteristic fragmentation patterns in the MS-spectra [83]. In most of the cases, the database proposals with highest similarity could therefore be used to score the candidate structure indirectly to one of the types. Candidate structures were excluded from the profile if they could not be scored unambiguously to types.

### In vitro biological activity

AChE inhibition and SERT affinity of the standardized alkaloid extracts were tested using published methods [21]. AChE activity was conducted using isolated acetylcholinesterase (*Electrophorus electricus*, Sigma, Germany) and SERT activity using homogenates of whole rat brains except cerebellum. Galanthamine and fluoxetine hydrochloride were used as positive standards in the AChE and SERT assays, respectively. Data were analysed with the software package GraFit 5 (Erithacus Software Ltd.). Activity values are means of three individual determinations each performed in triplicate. In an initial screening, AChE inhibition was defined as minimum 50% inhibition at a concentration of 1.0 μg ml^-1^. Subsequently IC_50_ values were determined for all extracts deemed active according the initial screening. IC_50_ values < 50 μg ml^-1^ was considered active for the analysis. SERT activity was defined as more than 85% binding of extract at 5 mg ml^-1^. Subsequently IC_50_ values were determined for all extracts deemed active according the initial screening. IC_50_ values < 50 μg ml^-1^ was considered active for the analysis. These activity levels were designed to reflect the observed level of activity in the present study and do not necessarily reflect levels of pharmacological relevance, but within the range of proposed ecological relevance [84]. SERT activity data were not determined for eight species of *Narcissus* and these samples were pruned from the phylogenetic trees in the correlation tests.

### Phylogenetic signal

We assessed the relationship of phylogeny to chemical diversity and biological activity by calculating the phylogenetic signal present for individual alkaloid types and types of biological activity. Each alkaloid type was coded as being either present (1) or absent (0) for each species. Likewise, for biological activity, species were scored for the presence or absence of AChE inhibition or SERT binding activity. Two of the alkaloid traits (belladine and cherylline) are found only in one species each, rendering calculation of phylogenetic signal for these traits meaningless, so they were not included in this part of the analysis.

To quantify phylogenetic signal we used the recently developed *D* metric [57], specifically developed to deal with discrete binary traits. *D* is calculated as follows:

(1) $$D=\frac{\left(s_{\mathrm{obs}}-{\bar{s}}_{b}\right)}{\left({\bar{s}}_{r}-{\bar{s}}_{b}\right)}$$

where *s*_*obs*_ is the observed number of changes in the binary trait (here, a chemical component) across the ultrametric phylogeny,${\bar{s}}_{r}$ is the mean number of changes generated from 1000 random permutations of the species values at the tips of the phylogeny, and ${\bar{s}}_{b}$ is the mean number of changes generated from 1000 simulations of the evolution for the character by a Brownian motion model of evolution with likelihood of change being specified as that which produces the same number of tip species with each character state as the observed pattern. A *D* value of 1 ($s_{\mathrm{obs}}={\bar{s}}_{r}$) indicates that the trait has evolved in a way that cannot be distinguished from a random manner (i.e., no phylogenetic signal), whilst a *D* value of 0 ($s_{\mathrm{obs}}={\bar{s}}_{b}$) indicates that the trait has evolved in a phylogenetically highly correlated manner. Estimation of whether *D* differs significantly from 1 or 0 is achieved by evaluating where the observed number of changes (*s*_*obs*_) fits within the distribution of the 1000 generated *s*_*r*_ and *s*_*b*_ values respectively. Thus if 95% or more values of *s*_*r*_ are greater than *s*_*obs*_ then P (*D* = 1) ≤ 0.05 and the trait is significantly more phylogenetically structured than random expectation. Calculation of *D* was carried out using the packages *caper *[85] and *ape *[86] in the *R* v2.14.0 framework [87].

We also quantified the relationship between overall chemical profile and phylogeny following an approach used to study the evolution of pheromone chemical diversity [57,58]. We constructed pairwise matrices; one of ultrametric phylogenetic distances (summed branch lengths) between species, and the other of chemical difference calculated as the binary squared Euclidean distance (i.e., the total number of alkaloid types that are absent in one taxon but present in another and *vice versa*). In addition to using all included species as terminal taxa, we also pruned the phylogenetic tree to genera and compared the resulting distance matrix to summed chemical profiles for each genus. In the case of polyphyletic genera both clades were retained. The correlation between phylogenetic distance and chemical difference was calculated using Mantel tests, with rows and columns of the distance matrix being randomly perturbed and the correlation coefficient recalculated 999 times to generate a null frequency distribution. These tests were performed using the program GenAlEx [88].

## Competing interests

The authors declare that they have no competing interests.

## Authors' contributions

NRØN conceived and designed the study, assembled the matrices, except *nad1*, conducted the phylogenetic analyses. NRØN prepared the manuscript with MRES and GIS. ANKJ and PM participated in conceiving the study. ANKJ and NRØN designed the study of biological activity and chemical diversity and interpreted the results with GIS. SBC participated in interpretation of the chemical data. TB, MM, NRAS, and GIS produced sequences, chemical, and bioactivity data and participated in interpreting the results. GP provided and aligned *nad1* sequences and participated in interpretation and discussion of the results of the phylogenetic analysis. MRES conducted the analyses for phylogenetic signal, and designed the analyses and interpreted the results with NRØN. AWM provided the classification scheme for Amaryllidoideae and participated in interpretation and discussion of the results of the phylogenetic analysis. JvS facilitated fieldwork in KwaZulu-Natal. All authors read and approved the final manuscript.

## Supplementary Material

Additional file 1 **Bayesian consensus tree with posterior probabilities and parsimony bootstrap consensus tree for the total evidence analysis and bootstrap consensus trees of individual regions.** Figures S1-S7. Click here for file

Additional file 2 Details of material included in the present study. Click here for file

Additional file 3 Primers used in this study for amplification and sequencing of four DNA regions in Amaryllidaceae subfamily Amaryllidoideae. Click here for file
