# Supplementary material for: Can phylogeny predict chemical diversity and potential medicinal activity of plants? A case study of amaryllidaceae
Source: BMC Evol Biol. 2012 Sep 14;12:182. doi: 10.1186/1471-2148-12-182 (PMC3499480; doi:10.1186/1471-2148-12-182)

Figure S1.
Total Evidence – Bayesian consensus

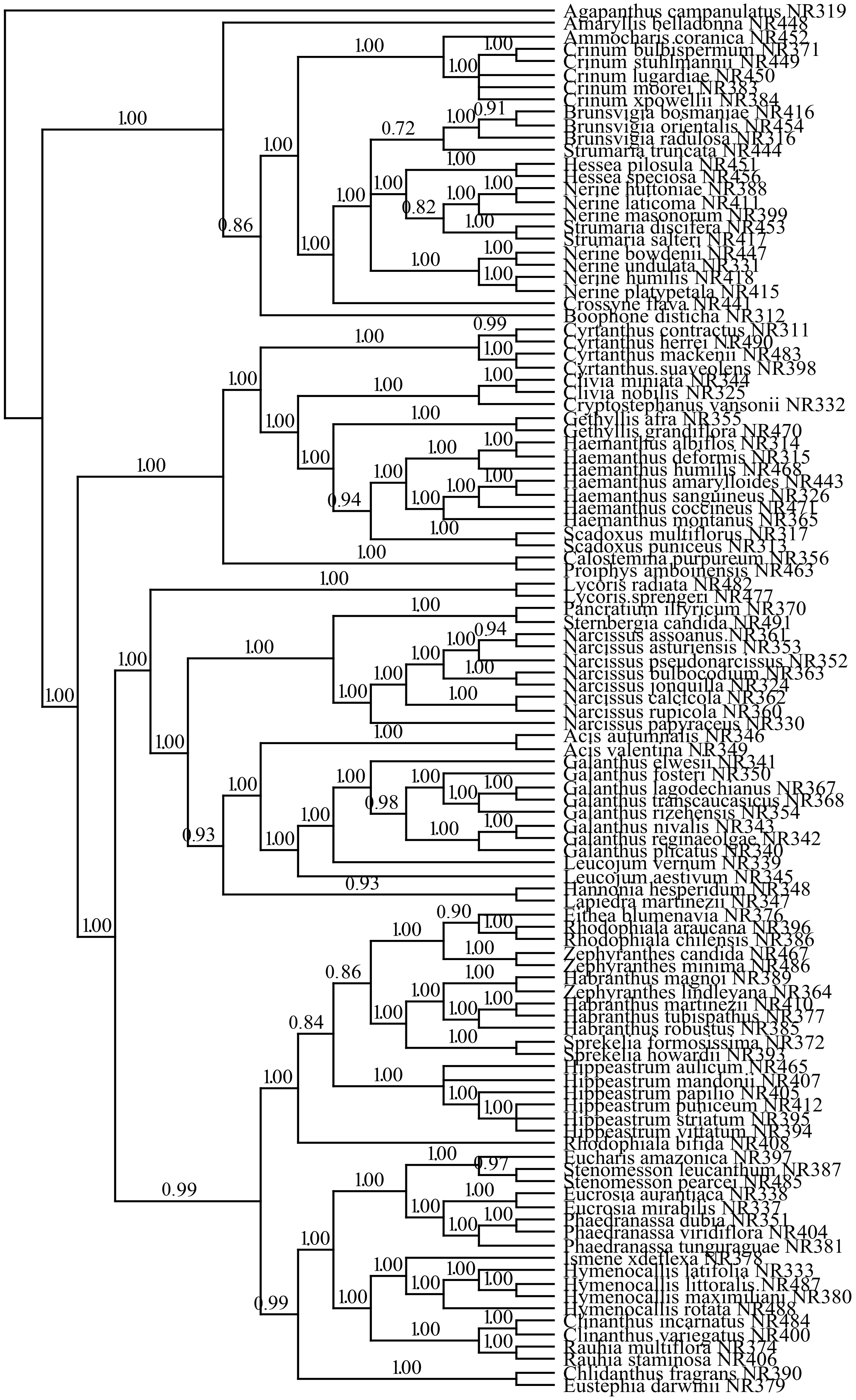

Figure S2.
Total Evidence - MP Bootstrap

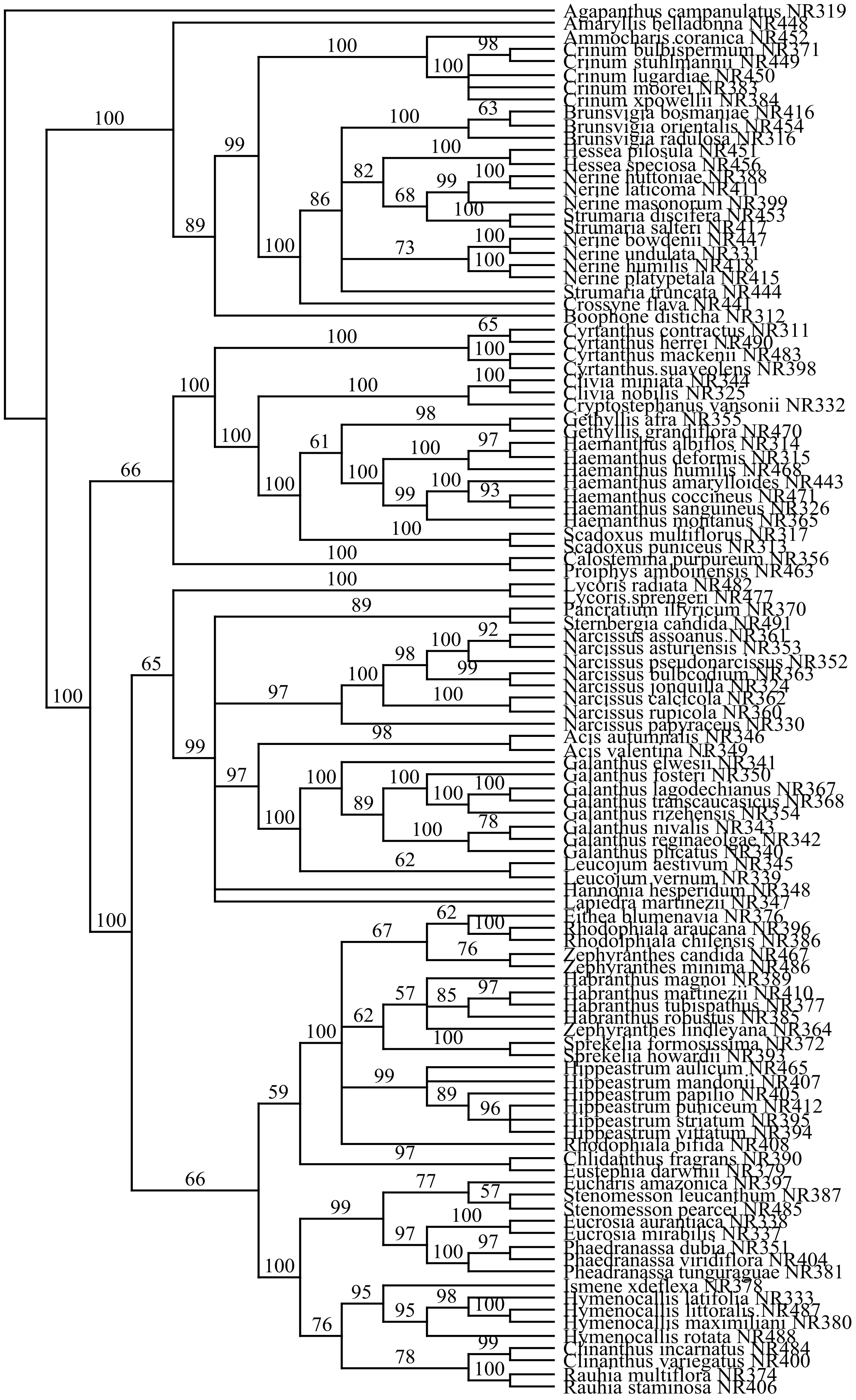

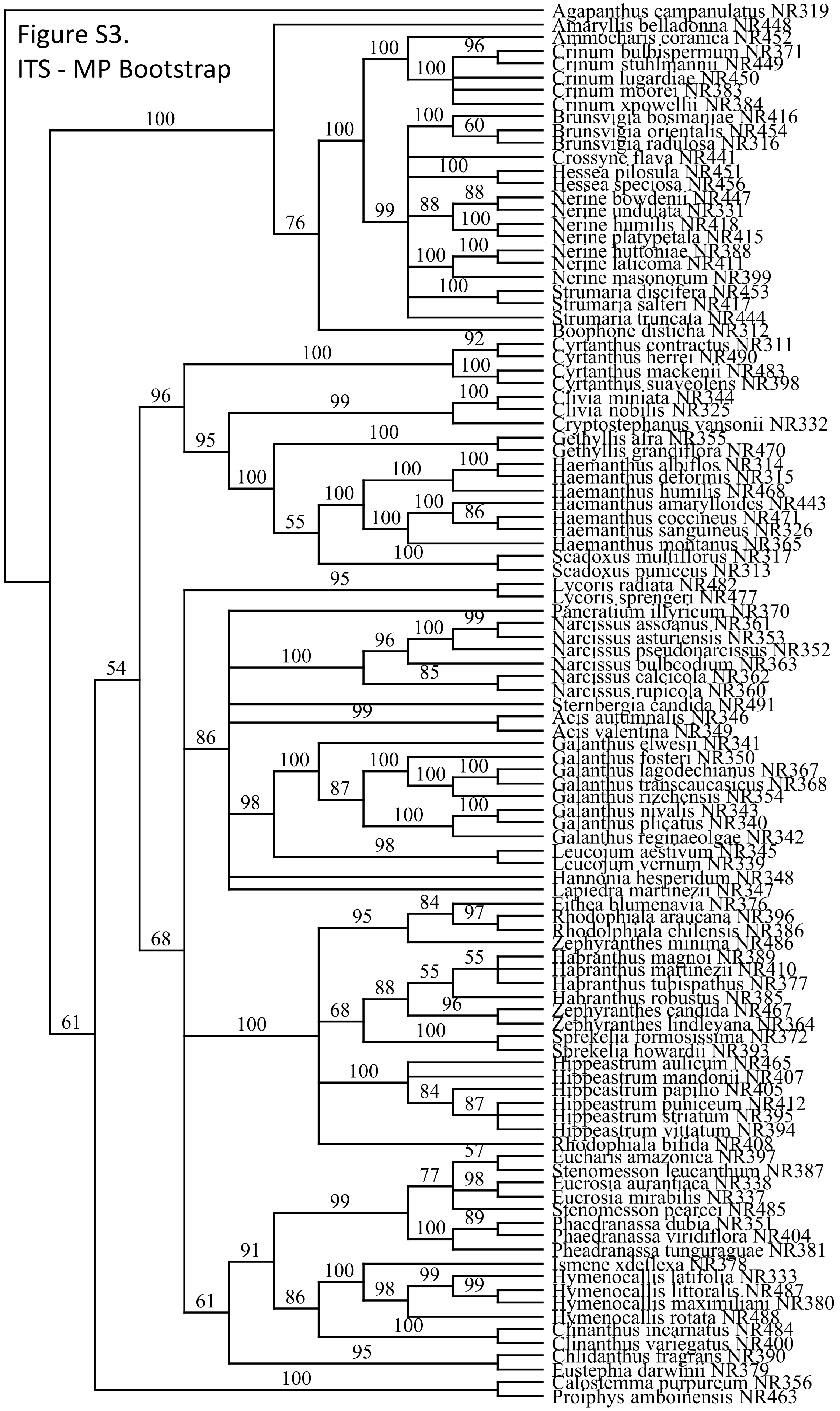

Figure S3.
ITS - MP Bootstrap

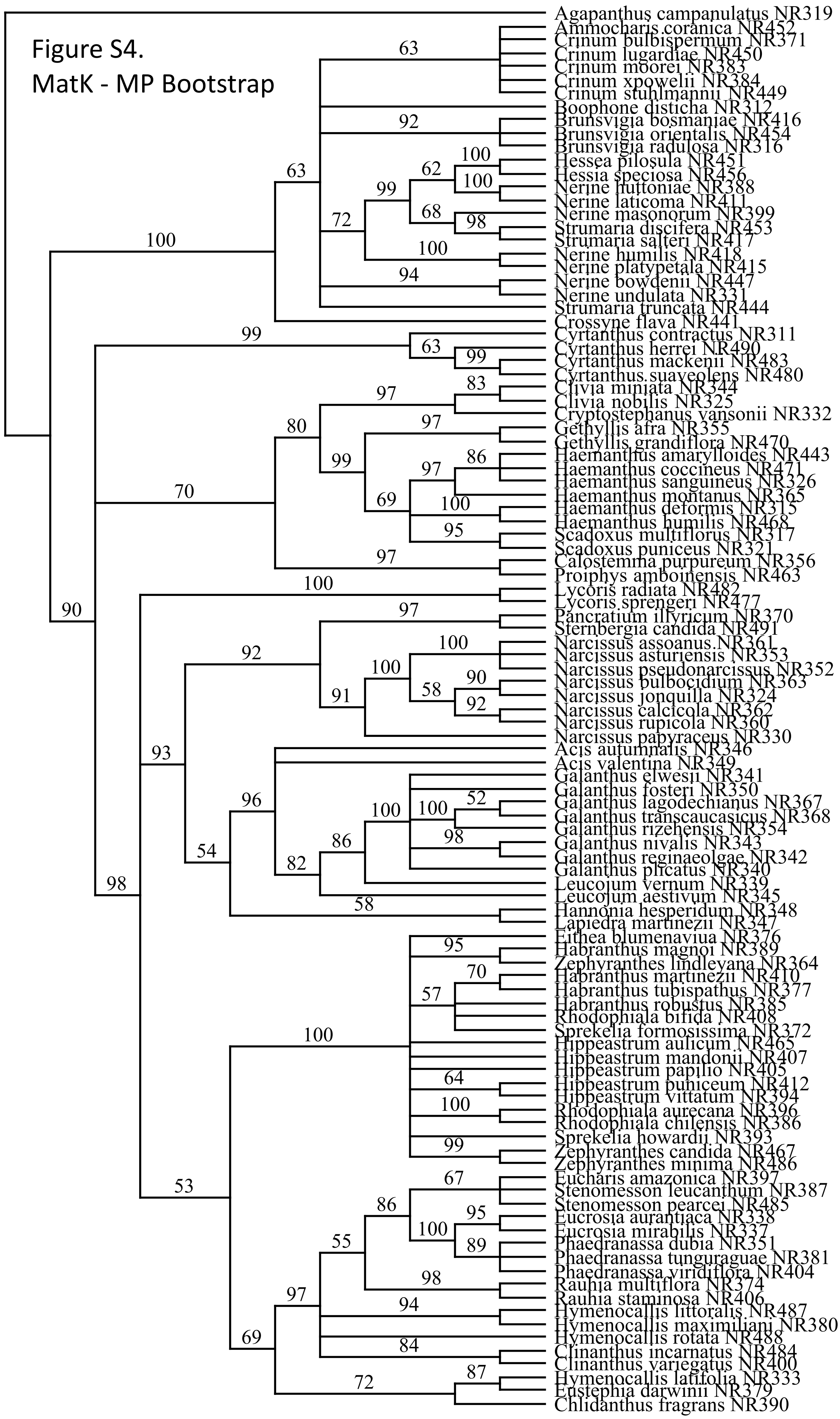
Figure S4.
MatK - MP Bootstrap
Agapanthus campanulatus NR319
Ammocharis coranica NR452
Crinum bulbispermum NR371
Crinum lugardiae NR450
Crinum moorei NR383
Crinum xpowelii NR384
Crinum stuhlmannii NR449
Boophone disticha NR312
Brunsvigia bosmaniae NR416
Brunsvigia orientalis NR454
Brunsvigia radulosa NR316
Hessea pilosula NR451
Hessia speciosa NR456
Nerine huttoniae NR388
Nerine laticoma NR411
Nerine masonorum NR399
Strumaria discifera NR453
Strumaria salteri NR417
Nerine humilis NR418
Nerine platypetala NR415
Nerine bowdenii NR447
Nerine undulata NR331
Strumaria truncata NR444
Crossyne flava NR441
Cyrtanthus contractus NR311
Cyrtanthus herrei NR490
Cyrtanthus mackenii NR483
Cyrtanthus suaveolens NR480
Clivia miniata NR344
Clivia nobilis NR325
Cryptostephanus vansonii NR332
Gethyllis afra NR355
Gethyllis grandiflora NR470
Haemanthus amaryllоides NR443
Haemanthus coccineus NR471
Haemanthus sanguineus NR326
Haemanthus montanus NR365
Haemanthus deformis NR315
Haemanthus humilis NR468
Scadoxus multiflorus NR317
Scadoxus puniceus NR321
Calostemma purpureum NR356
Proiphys amboinensis NR463
Lycoris radiata NR482
Lycoris sprengeri NR477
Pancratium illyricum NR370
Sternbergia candida NR491
Narcissus assoanus NR361
Narcissus asturiensis NR353
Narcissus pseudonarcissus NR352
Narcissus bulbocidium NR363
Narcissus jonquilla NR324
Narcissus calcicola NR362
Narcissus rupicola NR360
Narcissus papyraceus NR330
Acis autumnalis NR346
Acis valentina NR349
Galanthus elwesii NR341
Galanthus fosteri NR350
Galanthus lagodechianus NR367
Galanthus transcaucasicus NR368
Galanthus rizehensis NR354
Galanthus nivalis NR343
Galanthus reginaeolgae NR342
Galanthus plicatus NR340
Leucojum vernum NR339
Leucojum aestivum NR345
Hannonia hesperidum NR348
Lapiedra martinezii NR347
Eithea blumenaviua NR376
Habranthus magnoi NR389
Zephyranthes lindleyana NR364
Habranthus martinezii NR410
Habranthus tubispathus NR377
Habranthus robustus NR385
Rhodophiala bifida NR408
Sprekelia formosissima NR372
Hippeastrum aulicum NR465
Hippeastrum mandonii NR407
Hippeastrum papilio NR405
Hippeastrum puniceum NR412
Hippeastrum vittatum NR394
Rhodophiala aurecana NR396
Rhodophiala chilensis NR386
Sprekelia howardii NR393
Zephyranthes candida NR467
Zephyranthes minima NR486
Eucharis amazonica NR397
Stenomesson leucanthum NR387
Stenomesson pearcei NR485
Eucrosia aurantiaca NR338
Eucrosia mirabilis NR337
Phaedranassa dubia NR351
Phaedranassa tunguraguae NR381
Phaedranassa viridiflora NR404
Rauhia multiflora NR374
Rauhia staminosa NR406
Hymenocallis littoralis NR487
Hymenocallis maximiliani NR380
Hymenocallis rotata NR488
Clinanthus incarnatus NR484
Clinanthus variegatus NR400
Hymenocallis latifolia NR333
Eustephia darwinii NR379
Chlidanthus fragrans NR390

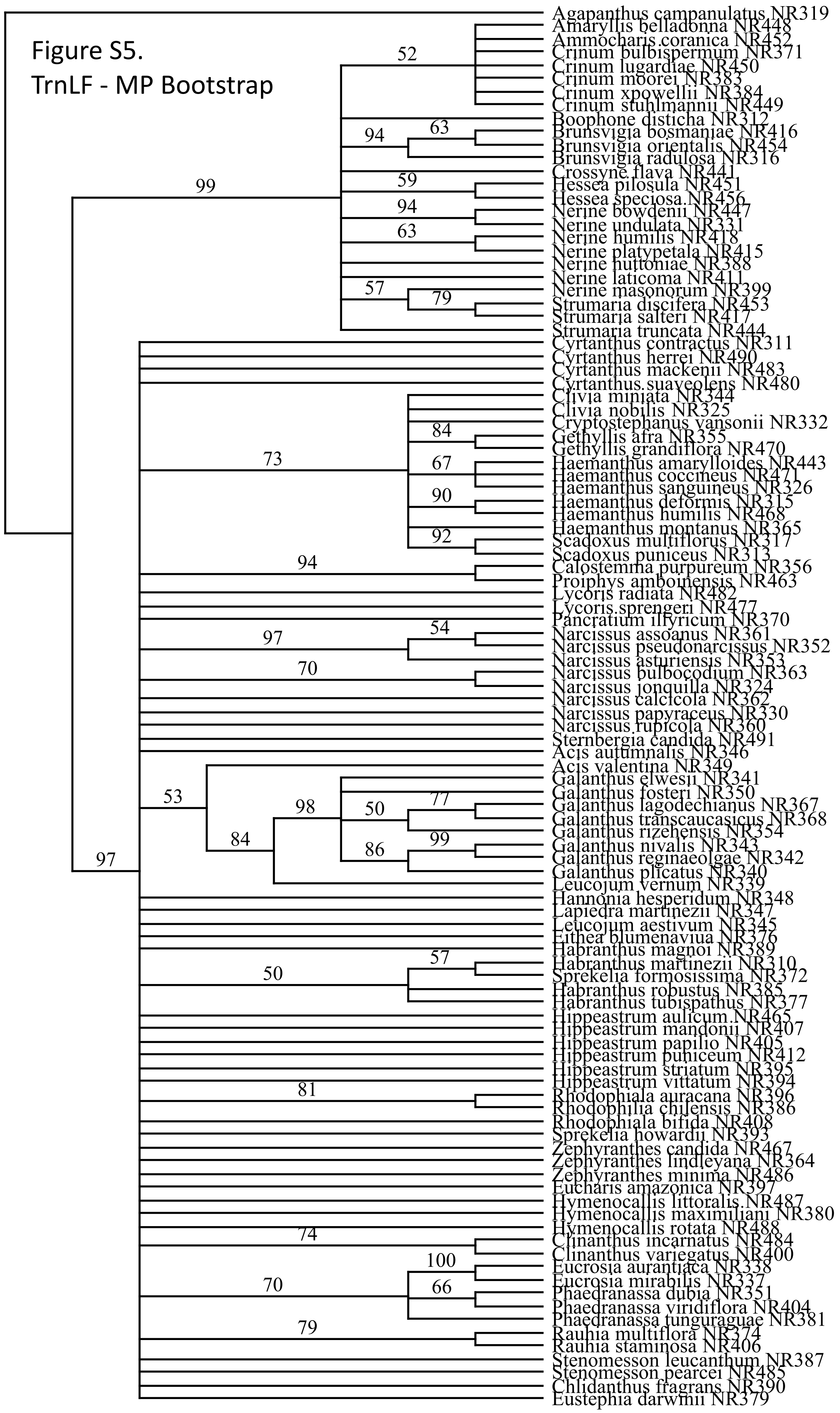
Figure S5.
TrnLF - MP Bootstrap
Agapanthus campanulatus NR319
Amaryllis belladonna NR448
Ammocharis coranica NR452
Crinum bulbispermum NR371
Crinum lugardiae NR450
Crinum moorei NR383
Crinum xpowellii NR384
Crinum stuhlmannii NR449
Boophone disticha NR312
Brunsvigia bosmaniae NR416
Brunsvigia orientalis NR454
Brunsvigia radulosa NR316
Crossyne flava NR441
Hessea pilosula NR451
Hessea speciosa NR456
Nerine bowdenii NR447
Nerine undulata NR331
Nerine humilis NR418
Nerine platypetala NR415
Nerine huttoniae NR388
Nerine laticoma NR411
Nerine masonorum NR399
Strumaria discifera NR453
Strumaria salteri NR417
Strumaria truncata NR444
Cyrtanthus contractus NR311
Cyrtanthus herrei NR490
Cyrtanthus mackenii NR483
Cyrtanthus suaveolens NR480
Clivia miniata NR344
Clivia nobilis NR325
Cryptostephanus vansonii NR332
Gethyllis afra NR355
Gethyllis grandiflora NR470
Haemanthus amarylloides NR443
Haemanthus coccineus NR471
Haemanthus sanguineus NR326
Haemanthus deformis NR315
Haemanthus humilis NR468
Haemanthus montanus NR365
Scadoxus multiflorus NR317
Scadoxus puniceus NR313
Calostemma purpureum NR356
Proiphys amboinensis NR463
Lycoris radiata NR482
Lycoris sprengeri NR477
Pancratium illyricum NR370
Narcissus assoanus NR361
Narcissus pseudonarcissus NR352
Narcissus asturiensis NR353
Narcissus bulbocodium NR363
Narcissus jonquilla NR324
Narcissus calcicola NR362
Narcissus papyraceus NR330
Narcissus rupicola NR360
Sternbergia candida NR491
Acis autumnalis NR346
Acis valentina NR349
Galanthus elwesii NR341
Galanthus fosteri NR350
Galanthus lagodechianus NR367
Galanthus transcaucasicus NR368
Galanthus rizehensis NR354
Galanthus nivalis NR343
Galanthus reginaeolgae NR342
Galanthus plicatus NR340
Leucojum vernum NR339
Hannonia hesperidum NR348
Lapiedra martinezii NR347
Leucojum aestivum NR345
Eithea blumenaviua NR376
Habranthus magnoi NR389
Habranthus martinezii NR310
Sprekelia formosissima NR372
Habranthus robustus NR385
Habranthus tubispathus NR377
Hippeastrum aulicum NR465
Hippeastrum mandonii NR407
Hippeastrum papilio NR405
Hippeastrum puniceum NR412
Hippeastrum striatum NR395
Hippeastrum vittatum NR394
Rhodophiala auracana NR396
Rhodophiala chilensis NR386
Rhodophiala bifida NR408
Sprekelia howardii NR393
Zephyranthes candida NR467
Zephyranthes lindleyana NR364
Zephyranthes minima NR486
Eucharis amazonica NR397
Hymenocallis littoralis NR487
Hymenocallis maximiliani NR380
Hymenocallis rotata NR488
Clinanthus incarnatus NR484
Clinanthus variegatus NR400
Eucrosia aurantiaca NR338
Eucrosia mirabilis NR337
Phaedranassa dubia NR351
Phaedranassa viridiflora NR404
Phaedranassa tunguraguae NR381
Rauhia multiflora NR374
Rauhia staminosa NR406
Stenomesson leucanthum NR387
Stenomesson pearcei NR485
Chlidanthus fragrans NR390
Eustephia darwinii NR379

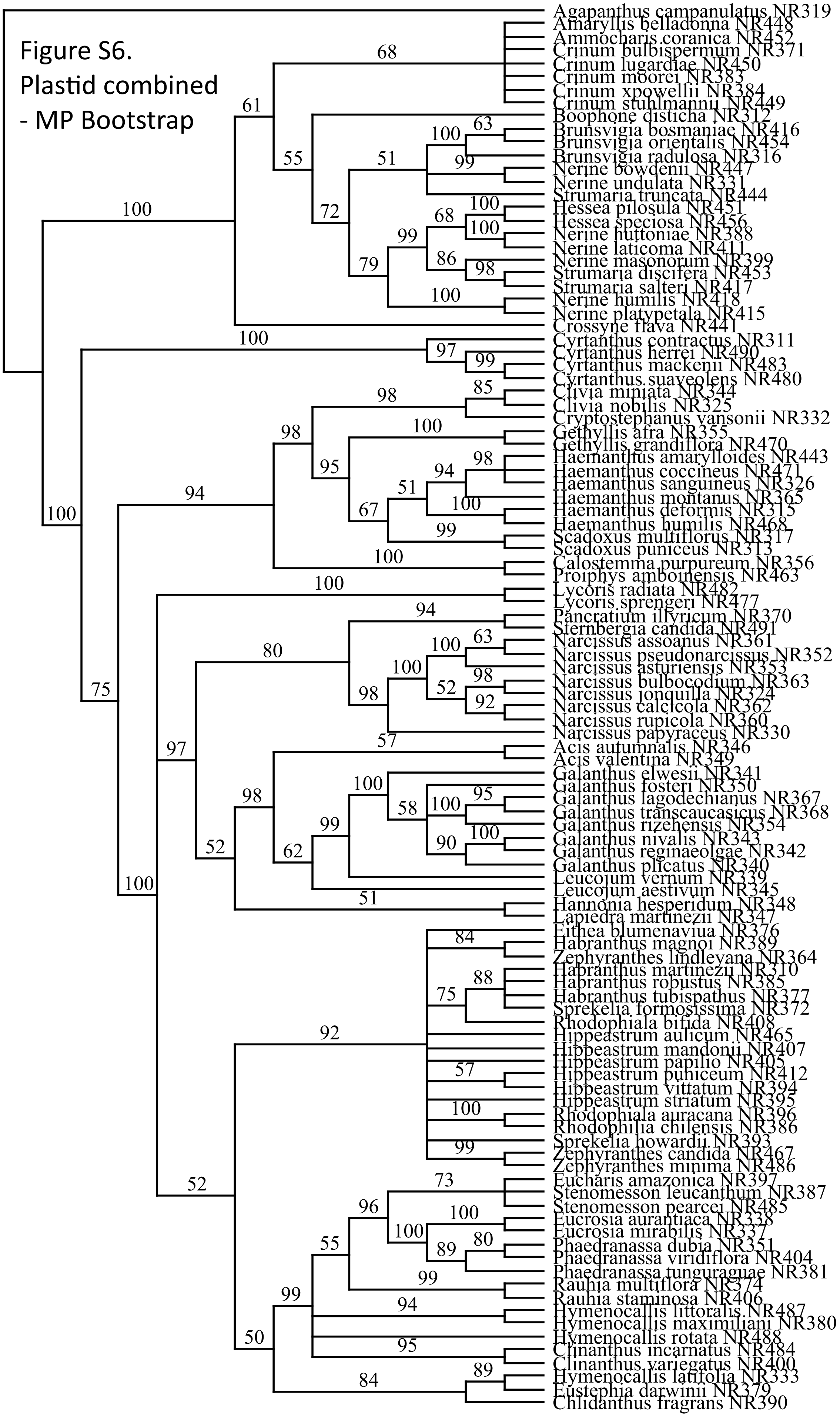

Figure S6.
Plastid combined
- MP Bootstrap

Figure S7.
Nad1 - MP Bootstrap

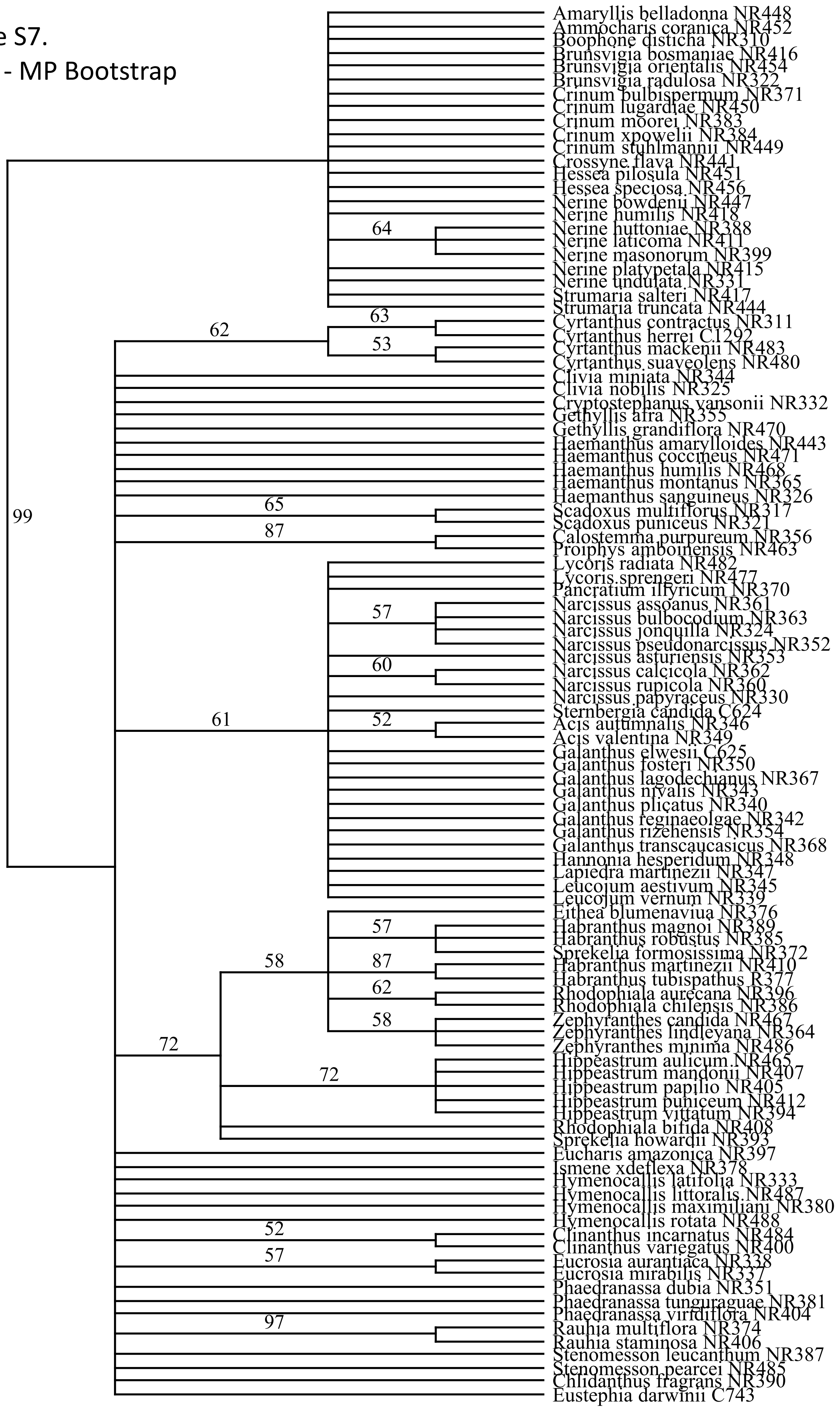

Supplement: Additional file 1 — Bayesian consensus tree with posterior probabilities and parsimony bootstrap consensus tree for the total evidence analysis and bootstrap consensus trees of individual regions. Figures S1-S7. [file 1471-2148-12-182-S1.pdf]
