# Supplementary material for: Can phylogeny predict chemical diversity and potential medicinal activity of plants? A case study of amaryllidaceae
Source: BMC Evol Biol. 2012 Sep 14;12:182. doi: 10.1186/1471-2148-12-182 (PMC3499480; doi:10.1186/1471-2148-12-182)
Supplement: Additional file 2 — Details of material included in the present study. [file 1471-2148-12-182-S2.docx]

**Additional File 2. Details of material included in the present study.**

| **Taxa** | **Voucher** | **Origin** | **ITS** | ***trnLF*** | ***matK*** | ***nad1*** | **Alkaloid types**^4^ | **AChE**^5^ | **SERT**^5^ |
| --- | --- | --- | --- | --- | --- | --- | --- | --- | --- |
| **Outgroup** | | | | | | | | | |
| *Agapanthus campanulatus* F.M.Leight | Rønsted 319 (C) | University of KZN | JX464256 | JX464333 | JX464521 | - | None | 0 | 0 |
| **Tribe Amaryllideae J. St.-Hil.** | | | | | | | | | |
| *Amaryllis belladonna* L. | Rønsted 448 (C) | RarePlants.co.uk, UK^1^ | JX464257 | JX464334 | - | JX464417 | 2,4 | 1 (16.2) | 0 |
| *Ammocharis coranica* (Ker Gawl.) Herb. | Rønsted 452 (C) | Telos Rare Bulbs, USA^1^ | JX464258 | JX464335 | JX464522 | JX464418 | 2,4 | 1 (10.4) | 0 |
| *Boophone disticha* (L.f.) Herb. | Rønsted 312 (C) | Mona Market, Northern Zululand, South Africa | JX464259 | JX464336 | JX464523 | JX464419 | 2 | 1 (12.8) | 0 |
| *Brunsvigia bosmaniae* F.M.Leight | Rønsted 416 (C) | Telos Rare Bulbs, USA^1^ | JX464260 | JX464337 | JX464524 | JX464420 | 2,4 | 1 (10.4) | 0 |
| *Brunsvigia orientalis* (L.) Aiton ex Eckl. | Rønsted 454 (C) | Telos Rare Bulbs, USA^1^ | JX464261 | JX464338 | JX464525 | JX464421 | 2,3,4,7 | 1 (12.9) | 0 |
| *Brunsvigia radulosa* Herb. | Rønsted 316 (C) | Midmar Dam, KZN | JX464262 | JX464339 | JX464526 | JX464422 | 4 | 0 | 0 |
| *Crinum bulbispermum* (Burm.f.) Milne-Redh. & Schweick | Rønsted 371 (C) | P1979-5384 (C) | JX464263 | JX464340 | JX464527 | JX464423 | 2,4 | 1 (4.3) | 0 |
| *Crinum lugardiae* N.E.Br. | Rønsted 450 (C) | Telos Rare Bulbs, USA^1^ | JX464264 | JX464341 | JX464528 | JX464424 | 4 | 1 (10.7) | 0 |
| *Crinum moorei* Hook.f. | Rønsted 383 (C) | E1276-0008 (C) | JX464265 | JX464342 | JX464529 | JX464425 | 2,4 | 1 (4.1) | 0 |
| *Crinum x powellii* Baker | Rønsted 384 (C) | E1276-0009B (C) | JX464266 | JX464343 | JX464530 | JX464426 | 2,4 | 1 (2.4) | 0 |
| *Crinum stuhlmannii* subsp. *delagoense* (I. Verd.) Kwembeya & Nordal | Rønsted 449 (C) | Telos Rare Bulbs, USA^1^ | JX464267 | JX464344 | JX464531 | JX464427 | 2,4 | 1 (5.0) | 0 |
| *Crossyne flava* (W.F.Barker ex Snijman) D.Müll.-Doblies & U.Müll.-Doblies | Rønsted 441 (C) | Telos Rare Bulbs, USA^1^ | JX464268 | JX464345 | JX464532 | JX464428 | 2,3,4,7 | 1 (29.0) | 0 |
| *Hessea pilosula* D.Müll.-Doblies & U.Müll.-Doblies | Rønsted 451 (C) | RarePlants.co.uk, UK^1^ | JX464269 | JX464346 | JX464533 | JX464429 | 2,4 | 1 (8.5) | 0 |
| *Hessea speciosa* Snijman | Rønsted 456 (C) | RarePlants.co.uk, UK^1^ | JX464270 | JX464347 | JX464534 | JX464430 | 2,4,6 | 1 (12.6) | 0 |
| *Nerine bowdenii* W.Watson | Rønsted 447 (C) | P2000-5013 (C) | JX464271 | JX464348 | JX464535 | JX464431 | 2 | 1 (23.1) | 0 |
| *Nerine humilis* (Jacq.) Herb | Rønsted 418 (C) | Telos Rare Bulbs, USA^1^ | JX464272 | JX464349 | JX464536 | JX464432 | 2,4,9 | 1 (7.2) | 0 |
| *Nerine huttoniae* Schönland | Rønsted 388 (C) | Telos Rare Bulbs, USA^1^ | JX464273 | JX464350 | JX464537 | JX464433 | 2,4,6 | 1 (8.8) | 0 |
| *Nerine laticoma* (Ker Gawl.) T.Durand & Schinz | Rønsted 411 (C) | Telos Rare Bulbs, USA^1^ | JX464274 | JX464351 | JX464538 | JX464434 | 4,9 | 1 (4.9) | 0 |
| *Nerine masonorum* L.Bolus | Rønsted 399 (C) | Telos Rare Bulbs, USA^1^ | JX464275 | JX464352 | JX464539 | JX464435 | 2,4,6 | 1 (13.5) | 0 |
| *Nerine platypetala* McNeil | Rønsted 415 (C) | Telos Rare Bulbs, USA^1^ | JX464276 | JX464353 | JX464540 | JX464436 | 1,2,4,6,9 | 1 (9.6) | 0 |
| *Nerine undulata* (L.) Herb. | Rønsted 331(C) | E1273C001A (C) | JX464277 | JX464354 | JX464541 | JX464437 | 2,4 | 1 (10.2) | 0 |
| *Strumaria discifera* Marloth ex Snijman | Rønsted 453 (C) | Telos Rare Bulbs, USA^1^ | JX464278 | JX464355 | JX464542 | - | 1,7 | 0 | 0 |
| *Strumaria salteri* W.F.Barker | Rønsted 417 (C) | Telos Rare Bulbs, USA^1^ | JX464279 | JX464356 | JX464543 | JX464438 | 2,9 | 1 (8.0) | 0 |
| *Strumaria truncata* Jacq. | Rønsted 444 (C) | Telos Rare Bulbs, USA^1^ | JX464280 | JX464357 | JX464544 | JX464439 | 4,6 | 1 (14.0) | 0 |
| **Tribe Cyrtantheae Salisb.** | | | | | | | | | |
| *Cyrtanthus contractus* N.E.Br. | Rønsted 311 (C) | KZN, South Africa | JX464281 | JX464358 | JX464545 | JX464440 | - | 0 | 0 |
| *Cyrtanthus herrei* (F.M.Leight) R.A.Dyer | Rønsted 490 (C) | S1997-0653 (C) | JX464282 | JX464359 | JX464546 | JX464441 | 2,4,9 | 1 (10.4) | 0 |
| *Cyrtanthus mackenii* Hook.f. | Rønsted 483 (C) | RarePlants.co.uk, UK^1^ | JX464283 | JX464360 | JX464547 | JX464442 | 3,4,7 | 0 | 0 |
| *Cyrtanthus suaveolens* Schönland | Rønsted 398 (C) | Telos Rare Bulbs, USA^1^ | JX464284 | JX464361 | JX464548 | JX464443 | - | 0 | 0 |
| **Tribe Haemantheae (Pax) Hutch.** | | | | | | | | | |
| *Clivia miniata* (Lindl.) Bosse | Rønsted 344 (C) | E1291−0002 (C) | HM140798^2^ | HM140815^2^ | JX464549 | JX464444 | 3,4,6 | 1 (18.0) | 0 |
| *Clivia nobilis* Lindl. | Rønsted 325 (C) | E1291−0001 (C) | HM140799^2^ | JX464362 | JX464550 | JX464445 | 3,4,6 | 1 (41.0) | 0 |
| *Cryptostephanus vansonii* Verd. | Rønsted 332 (C) | S2002−0104 (C) | HM140800^2^ | JX464363 | JX464551 | JX464446 | 2,3,5,7,8 | 1 (28.0) | 0 |
| *Gethyllis afra* L. | Rønsted 355 (C) | Telos Rare Bulbs, USA^1^ | HM140801^2^ | JX464364 | JX464552 | JX464447 | - | 0 | 0 |
| *Gethyllis grandiflora* L.Bolus | Rønsted 470 (C) | RarePlants.co.uk, UK^1^ | HM140805^2^ | JX464365 | JX464553 | JX464448 | - | 0 | 0 |
| *Haemanthus albiflos* Jacq. | Rønsted 314 (C) | University of KZN | HM140803^2^ | - | - | - | 6,7 | 0 | 0 |
| *Haemanthus amarylloides* Jacq. | Rønsted 443 (C) | Telos Rare Bulbs, USA^1^ | HM140804^2^ | HM140816^2^ | JX464554 | JX464449 | 3,4,6,7 | 1 (33.0) | 0 |
| *Haemanthus coccineus* L. | Rønsted 471 (C) | RarePlants.co.uk, UK^1^ | HM140805^2^ | JX464366 | JX464555 | JX464450 | 2,7 | 1 (43.0) | 1 (8.7) |
| *Haemanthus deformis* Hook.f. | Rønsted 315 (C) | Midmar Dam, KZN, SA | HM140807^2^ | HM140818^2^ | JX464556 | - | 2,6,7 | 0 | 0 |
| *Haemanthus humilis* subsp. *hirsutus* (Baker) Snijman | Rønsted 468 (C) | RarePlants.co.uk, UK^1^ | HM140808^2^ | HM140819^2^ | JX464557 | JX464451 | 4,6,7 | 1 (25.0) | 1 (1.7) |
| *Haemanthus montanus* Baker | Rønsted 365 (C) | RarePlants.co.uk, UK^1^ | HM140809^2^ | HM140820^2^ | JX464558 | JX464452 | 2,6,7 | 1 (46.0) | 0 |
| *Haemanthus sanguineus* Jacq. | Rønsted 326 (C) | P1999–5533 (C) | HM140810^2^ | HM140821^2^ | JX464559 | JX464453 | 2,3,7,9 | 1 (8.0) | 0 |
| *Scadoxus multiflorus* (Martyn) Raf. | Rønsted 317 (C) | Andrew James Nursery, KZN^1^ | HM140812^2^ | HM140823^2^ | JX464560 | JX464454 | - | 0 | 0 |
| *Scadoxus puniceus* (L.) Friis & Nordal | Rønsted 313 (C) | Ngoye forest, KZN | HM140814^2^ | HM140825^2^ | JX464561 | JX464455 | 2,4,7,9 | 1 (18.0) | 0 |
| **Tribe Calostemmateae D. & U. M.-D.** | | | | | | | | | |
| *Calostemma purpureum* R.Br. | Rønsted 356 (C) | Telos Rare Bulbs, USA^1^ | JX464285 | JX464367 | JX464562 | JX464456 | 4,6,9 | 1 (9.2) | 1 (34.2) |
| *Proiphys amboinensis* (L.). Herb. | Rønsted 463 (C) | Tropical-Plants-Hub, Thailand^1^ | JX464286 | JX464368 | JX464563 | JX464457 | 2,4,6,9 | 1 (6.2) | 0 |
| **Tribe Lycoridae Traub** | | | | | | | | | |
| *Lycoris radiata* (L'Hér.) Herb. | Rønsted 482 (C) | RarePlants.co.uk, UK^1^ | JX464287 | JX464369 | JX464564 | JX464458 | 2,4,7 | 1 (26.4) | 0 |
| *Lycoris sprengeri* Comes ex Baker | Rønsted 477 (C) | RV Roger, UK^1^ | JX464288 | JX464370 | JX464565 | JX464459 | 3,4,6 | 1 (19.2) | 0 |
| **Tribe Pancratieae Salisb.** | | | | | | | | | |
| *Pancratium illyricum* L. | Rønsted 370 (C) | 1288-2 (C) | JX464289 | JX464371 | JX464566 | JX464460 | 2,3,5 | 1 (23.4) | 0 |
| **Tribe Narcisseae Endl.** | | | | | | | | | |
| *Narcissus assoanus* var. *assoanus* Dufour ex Schult & Schult | Rønsted 361 (C) | S1969-1395 (C) | JX464290 | JX464372 | JX464567 | JX464461 | - | 0 | - |
| *Narcissus asturiensis* (Jord.) Pugsley | Rønsted 353 (C) | P1997-5203 (C) | JX464291 | JX464373 | JX464568 | JX464462 | 3,4,6 | 0 | - |
| *Narcissus bulbcodium* L. | Rønsted 363 (C) | P1976-5516 (C) | JX464292 | JX464374 | JX464569 | JX464463 | - | 0 | - |
| *Narcissus calcicola* Mendonça | Rønsted 362 (C) | P1997-5204 (C) | JX464293 | JX464375 | JX464570 | JX464464 | 4 | 0 | - |
| *Narcissus jonquilla* L. | Rønsted 324 (C) | P1975-5871 (C) | - | JX464376 | JX464571 | JX464465 | 2,3,6,9 | 1 (8.7) | - |
| *Narcissus papyraceus* ssp. *papyraceus* Ker Gawl. | Rønsted 330 (C) | E1289-0042B (C) | - | JX464377 | JX464572 | JX464466 | 2,4,9 | 0 | - |
| *Narcissus pseudonarcissus* L. | Rønsted 352 (C) | P1992-5620B (C) | JX464294 | JX464378 | JX464573 | JX464467 | 2,3,6,9 | 1 (8.4) | - |
| *Narcissus rupicola* Dufour | Rønsted 360 (C) | 1289-26 (C) | JX464295 | JX464379 | JX464574 | JX464468 | - | 0 | - |
| *Sternbergia candida* B.Mathew & T.Baytop | Rønsted 491 (C) | 1993-5059 (C) | JX464296 | JX464380 | JX464575 | JX464469 | - | 0 | 0 |
| **Tribe Galantheae Salisb.** | | | | | | | | | |
| *Acis autumnalis* (L.) Sweet | Rønsted 346 (C) | Broadleigh Gard., UK^1^ | FN663865^3^ | FN663905^3^ | FN663885^3^ | JX464470 | 4 | 0 | 0 |
| *Acis valentina* (Pau) Lledó, A.P.Davis & M.B.Crespo | Rønsted 349 (C) | Broadleigh Gard., UK^1^ | FN663866^3^ | FN663911^3^ | FN663886^3^ | JX464471 | 3 | 1 (11.7) | 0 |
| *Galanthus elwesii* Hook.f. | Rønsted 341 (C) | 1265-0003C (C) | FN663870^3^ | FN663915^3^ | FN663889^3^ | JX464472 | 2,3,6,9 | 0 | 0 |
| *Galanthus fosteri* Baker | Rønsted 350 (C) | Broadleigh Gard., UK^1^ | FN663871^3^ | FN663916^3^ | FN663890^3^ | JX464473 | 4,9 | 0 | 0 |
| *Galanthus lagodechianus* Kem.-Nath. | Soelberg 367 (ERCB) | Yerevan Bot. Gard., Armenia | FN663873^3^ | FN663918^3^ | FN663892^3^ | JX464474 | - | 0 | 0 |
| *Galanthus nivalis* L. | Rønsted 343 (C) | 1265-0001C (C) | FN663874^3^ | FN663919^3^ | FN663893^3^ | JX464475 | - | 0 | 0 |
| *Galanthus plicatus* M.Bieb. | Rønsted 340 (C) | 0000-5242 (C) | FN663875^3^ | FN663940^3^ | FN663894^3^ | JX464476 | 4,9 | 0 | 0 |
| *Galanthus reginae-olgae* Orph. | Rønsted 342 (C) | 1992-5586, (C) | FN663878^3^ | FN663923^3^ | FN663897^3^ | JX464477 | 4,9 | 0 | 0 |
| *Galanthus rizehensis* Stern. | Rønsted 354 (C) | 1992-5591 (C) | FN663879^3^ | FN663924^3^ | FN663898^3^ | JX464478 | 4 | 0 | 0 |
| *Galanthus transcaucasicus* Fomin | Soelberg 368 (ERCB) | Shikakhogh Reserve, Armenia | FN663867^3^ | FN663912^3^ | FN663887^3^ | JX464479 | - | 0 | 0 |
| *Hannonia hesperidum* Braun-Blanq. & Maire | Rønsted 348 (C) | RarePlants.co.uk, UK^1^ | FN663880^3^ | FN663925^3^ | FN663899^3^ | JX464480 | 3,4,6 | 0 | 0 |
| *Lapiedra martinezii* Lag. | Rønsted 347 (C) | Telos Rare Bulbs, USA^1^ | FN663881^3^ | JX464381 | FN663900^3^ | JX464481 | 4,6,9 | 1 (9.3) | 0 |
| *Leucojum aestivum* L. | Rønsted 345 (C) | (C) Hort. | FN663882^3^ | FN663926^3^ | FN663901^3^ | JX464482 | 4 | 1 (17.8) | 0 |
| *Leucojum vernum* L. | Rønsted 339 (C) | 1266-0002 (C) | FN663883^3^ | FN663927^3^ | FN663902^3^ | JX464483 | 3,4 | 1 (26.5) | 0 |
| **Tribe Hippeastreae (Pax & Hoffmann) Hutch.** | | | | | | | | | |
| *Eithea blumenavia* (K.Koch & C.D.Bouché ex Carrière) Ravenna | Rønsted 376 (C) | Telos Rare Bulbs, USA^1^ | JX464297 | JX464382 | JX464576 | JX464484 | 2,3,4,6 | 1 (21.5) | 0 |
| *Habranthus magnoi* Ravenna | Rønsted 389 (C) | Telos Rare Bulbs, USA^1^ | JX464298 | JX464383 | JX464577 | JX464485 | - | 0 | 0 |
| *Habranthus martinezii* Ravenna | Rønsted 310 (C) | Telos Rare Bulbs, USA^1^ | JX464299 | JX464384 | JX464578 | JX464486 | 4 | 1 (42.7) | 0 |
| *Habranthus robustus* Herb. ex Sweet | Rønsted 385 (C) | Telos Rare Bulbs, USA^1^ | JX464300 | JX464385 | JX464579 | JX464487 | 3,4,6 | 0 | 0 |
| *Habranthus tubispathus* (L'Hér.) Traub | Rønsted 377 (C) | Telos Rare Bulbs, USA^1^ | JX464301 | JX464386 | JX464580 | JX464488 | 2,7,9 | 0 | 0 |
| *Hippeastrum aulicum* (Ker Gawl.) Herb | Rønsted 465 (C) | Telos Rare Bulbs, USA^1^ | JX464302 | NJX464387 | JX464581 | JX464489 | 2,4,6,9 | 1 (29.6) | 0 |
| *Hippeastrum mandonii* Baker | Rønsted 407 (C) | Telos Rare Bulbs, USA^1^ | JX464303 | JX464388 | JX464582 | JX464490 | 2,3,4 | 1 (23.9) | 0 |
| *Hippeastrum papilio* (Ravenna) Van Scheepen | Rønsted 405 (C) | RV Roger, UK^1^ | JX464304 | JX464389 | JX464583 | JX464491 | 2,3,6 | 1 (2.2) | 0 |
| *Hippeastrum puniceum* (Lam.) Voss | Rønsted 412 (C) | RV Roger, UK^1^ | JX464305 | JX464390 | JX464584 | JX464492 | 2,3,5 | 1 (23.6) | 0 |
| *Hippeastrum striatum* (Lam.) H.E.Moore | Rønsted 395 (C) | Telos Rare Bulbs, USA^1^ | JX464306 | JX464391 | - | - | - | 0 | 0 |
| *Hippeastrum vittatum* (L'Hér.) Herb. | Rønsted 394 (C) | RV Roger, UK^1^ | JX464307 | JX464392 | JX464585 | JX464493 | - | 0 | 0 |
| *Rhodophiala araucana* (Phil.) Traub | Rønsted 396 (C) | Telos Rare Bulbs, USA^1^ | JX464308 | JX464393 | JX464586 | JX464494 | 3,4,6,7,9 | 1 (27.5) | 0 |
| *Rhodophiala bifida* (Herb.) Traub | Rønsted 408 (C) | Telos Rare Bulbs, USA^1^ | JX464309 | JX464394 | JX464587 | JX464495 | 2,7,9 | 0 | 1 (7.4) |
| *Rhodophilia chilensis* (L'Hér.) Traub | Rønsted 386 (C) | Telos Rare Bulbs, USA^1^ | JX464310 | JX464395 | JX464588 | JX464496 | 2,3,4,6,7,9 | 1 (32.3) | 0 |
| *Sprekelia formosissima* (L.) Herb. | Rønsted 372 (C) | E1273-G001 (C) | JX464311 | JX464396 | JX464589 | JX464497 | 9 | 0 | 0 |
| *Sprekelia howardii* Lehmiller | Rønsted 393 (C) | Telos Rare Bulbs, USA^1^ | JX464312 | JX464397 | JX464590 | JX464498 | 3,4 | 1 (32.4) | 0 |
| *Zephyranthes candida* (Lindl.) Herb | Rønsted 467 (C) | Blomsterliv, DK^1^ | JX464313 | JX464398 | JX464591 | JX464499 | 2,3,4,6,9 | 1 (37.4) | 0 |
| *Zephyranthes lindleyana* Herb. | Rønsted 364 (C) | Telos Rare Bulbs, USA^1^ | JX464314 | JX464399 | JX464592 | JX464500 | 3,4 | 0 | 0 |
| *Zephyranthes minima* Herb. | Rønsted 486 (C) | RarePlants.co.uk, UK^1^ | JX464315 | JX464400 | JX464593 | JX464501 | 3 | 1 (30.3) | 0 |
| **Tribe Hymenocallideae (D. & U.M.-D.) Meerow** | | | | | | | | | |
| *Ismene x deflexa* Herb. | Rønsted 378 (C) | RarePlants.co.uk, UK^1^ | JX464316 | - | - | JX464502 | 3,6,9 | 0 | 0 |
| *Hymenocallis latifolia* (Mill.) M.Roem | Rønsted 333 (C) | P1891-5087 (C) | JX464317 | - | JX464594 | JX464503 | 2,3,9 | 1 (5.7) | 0 |
| *Hymenocallis littoralis* (Jacq.) Salisb. | Rønsted 487 (C) | Telos Rare Bulbs, USA^1^ | JX464318 | JX464401 | JX464595 | JX464504 | 2,3,5,9 | 1 (18.2) | 0 |
| *Hymenocallis maximiliani* T.M.Howard | Rønsted 380 (C) | Telos Rare Bulbs, USA^1^ | JX464319 | JX464402 | JX464596 | JX464505 | 2,3,9 | 1 (20.9) | 0 |
| *Hymenocallis rotata* (Ker Gawl.) Herb. | Rønsted 488 (C) | Telos Rare Bulbs, USA^1^ | JX464320 | JX464403 | JX464597 | JX464506 | 9 | 1 (34.6) | 0 |
| **Tribe Clinantheae Meerow** | | | | | | | | | |
| *Clinanthus incarnatus* (Kunth) Meerow | Rønsted 484 (C) | Telos Rare Bulbs, USA^1^ | JX464321 | JX464404 | JX464598 | JX464507 | 2,4 | 1 (22.6) | 1 (3.4) |
| *Clinanthus variegatus* (Ruiz & Pav.) Meerow | Rønsted 400 (C) | Telos Rare Bulbs, USA^1^ | JX464322 | JX464405 | JX464599 | JX464508 | 2,4 | 0 | 1 (28.3) |
| **Tribe Eucharideae (Pax) Hutch.** | | | | | | | | | |
| *Eucharis amazonica* Linden ex Planch | Rønsted 397 (C) | RarePlants.co.uk, UK^1^ | JX464323 | JX464406 | JX464600 | JX464509 | 3,4 | 1 (9.3) | 0 |
| *Eucrosia aurantiaca* (Baker) Pax | Rønsted 338 (C) | Telos Rare Bulbs, USA^1^ | JX464324 | JX464407 | JX464601 | JX464510 | 2,3,9 | 1 (4.1) | 0 |
| *Eucrosia mirabilis* (Baker) Pax | Rønsted 337 (C) | Telos Rare Bulbs, USA^1^ | JX464325 | JX464408 | JX464602 | JX464511 | 3 | 0 | 1 (18.4) |
| *Phaedranassa dubia* (Kunth) J.F.Macbr. | Rønsted 351 (C) | Telos Rare Bulbs, USA^1^ | FN663884^3^ | FN663928^3^ | FN663904^3^ | JX464512 | 2,3,4,6 | 1 (14.2) | 0 |
| *Phaedranassa tunguraguae* Ravenna | Rønsted 381 (C) | Telos Rare Bulbs, USA^1^ | JX464327 | JX464409 | JX464603 | JX464513 | 3,4,6 | 1 (11.0) | 0 |
| *Phaedranassa viridiflora* Regel | Rønsted 404 (C) | Telos Rare Bulbs, USA^1^ | JX464328 | JX464410 | JX464604 | JX464514 | 2,3,4,9 | 1 (37.5) | 0 |
| *Rauhia multiflora* (Kunth) Ravenna | Rønsted 374 (C) | P1985-5240 (C) | - | JX464411 | JX464605 | JX464515 | 2,3,7 | 1 (1.3) | 0 |
| *Rauhia staminosa* Ravenna | Rønsted 406 (C) | Telos Rare Bulbs, USA^1^ | - | JX464412 | JX464606 | JX464516 | 2,3,4,5,9 | 1 (0.4) | 0 |
| *Stenomesson leucanthum* (Ravenna) Meerow and van der Werff | Rønsted 387 (C) | Telos Rare Bulbs, USA^1^ | JX464329 | JX464413 | JX464607 | JX464517 | 6,9 | 0 | 0 |
| *Stenomesson pearcei* Baker | Rønsted 485 (C) | Telos Rare Bulbs, USA^1^ | JX464330 | JX464414 | JX464608 | JX464518 | 2,3,9 | 1 (11.3) | 0 |
| **Tribe Eustephiae (Pax) Hutch.** | | | | | | | | | |
| *Chlidanthus fragrans* Herb. | Rønsted 390 (C) | Blomsterliv, DK^1^ | JX464331 | JX464415 | JX464609 | JX464519 | 2,3,4,9 | 1 (18.0) | 0 |
| *Eustephia darwinii* Vargas | Rønsted 379 (C) | Telos Rare Bulbs, USA^1^ | JX464332 | JX464416 | JX464610 | JX464520 | 2,3,6,9 | 1 (9.6) | 0 |

Names according to the World Check list on Amaryllidaceae (Govaerts et al., 2007). (C) Herbarium C, the Natural History Museum of Denmark. ^1^Nursery. ^2^Bay-Smidt *et al.*, 2011. ^3^Larsen *et al.*, 2010. ^4^Alkaloid types (Jin, 2009): 1 = Belladine; 2 = Crinine; 3 = Galanthamine; 4 = Lycorine; 5 = Galanthindole; 6 = Homolycorine; 7 = Montanine; 8 = Cherylline; 9 = Tazettine. ^5^In vitro activity. Inhibition of acetylcholinesterase (AChE) and binding to the serotonin reuptake transporter (SERT) were used as proxies for biological activity. Biological activity was defined as an IC_50_ value above 50 μg/ml (given in parentheses, μg/ml).
