# Supplementary material for: Can phylogeny predict chemical diversity and potential medicinal activity of plants? A case study of amaryllidaceae
Source: BMC Evol Biol. 2012 Sep 14;12:182. doi: 10.1186/1471-2148-12-182 (PMC3499480; doi:10.1186/1471-2148-12-182)
Supplement: Additional file 3 — Primers used in this study for amplification and sequencing of four DNA regions in Amaryllidaceae subfamily Amaryllidoideae. [file 1471-2148-12-182-S3.pdf]

Additional file 3. Primers used in this study for amplification and sequencing of four DNA regions in Amaryllidaceae subfamily Amaryllidoideae.

Region	Primer	Sequence 5'-3'	Direction	Reference
ITS	ITS5	GGA AGT AAA AGT CGT AAC AAG G	Forward	White *et al.* (1999)
	ITS4	TCC TCC GCT TAT TGA TAT GC	Reverse	White *et al.* (1999)
	ITS1[1]	TCC GTA GGT GAA CCT GCG G	Forward	White *et al.* (1999)
	ITS2[1]	GCT GCG TTC TTC ATC GAT GC	Reverse	White *et al.* (1999)
trnLF	trnC	CGA AAT CGG TAG ACG CTA CG	Forward	Taberlet *et al.* (1991)
	trnF	ATT TGA ACT GGT GAC ACG AG	Reverse	Taberlet *et al.* (1991)
matK	19F	CGT TCT GAC CAT ATT GCA CTA TG	Forward	Kores *et al.* (2000)
	2R	AAC TAG TCG GAT GGA GTA G	Reverse	Johnson & Soltis (1996)
	1326R[1]	TCT AGC ACA CGA AAG TCG AAG T	Reverse	Sun *et al.* (2001)
	KatF[1]	ATC GGG CCA TCC TAT TAG	Forward	Larsen *et al.* (2010)
nad1	nad1eB	GCA TTA CGA TCT GCA GCT CA	Forward	Demesure *et al.* (1995)

	nad1eCR	GGA GCT CGA TTA GTT TCT GC	Reverse	Demesure *et al.* (1995)
	nad1iB2[1]	GTC GAG CAT ACG ACG ATG CCG C	Forward	Cuenca *et al.* (in press)
	nad1iB2R [1]	GGC GGC ATC GTC GTA TGC TCG	Reverse	Cuenca *et al.* (in press)

[1]Primarily used for sequencing.

Cuenca A, Petersen G, Seberg O. 2012. Genes and processed paralogs coexist in plant mitochondria. *Journal of Molecular Evolution* (in press).

Demesure B, Sodzi N, Petit RJ. 1995. A set of universal primers for amplification of polymorphic non-coding regions of mitochondrial and chloroplast DNA in plants. *Molecular Ecology* **4**: 129-134.

Johnson LA, Soltis DE. 1995. *matK* DNA sequences and phylogenetic reconstruction in Saxifragaceae s.s. *Systematic Botany* **75**: 753-766.

Kores PJ, Weston PH, Molvray M, Chase MW. 2000. Phylogenetic relationships within the Diurideae (Orhidaceae): inferences from plastid *matK* DNA sequences. In: Wilson KL, Morrison, DA, eds. *Monocots: Systematics and Evolution.* CSIRO Publishing, Collingwood, Victoria, Australia, pp. 449–456.

Larsen MM, Adsersen AA, Davis AP, Lledó MD, Jäger AK, Rønsted N. 2010. Using a phylogenetic approach to selection of target plants in drug discovery of acetylcholinesterase inhibiting alkaloids in Amaryllidaceae tribe Galantheae. *Biochemical Systematics and Ecology* **38**: 1026-1034.

Sun H, McLewin W, Fay MF. 2001. Molecular phylogeny of *Helleborus* (Ranunculaceae), with an emphasis on the east Asian-Mediterranean disjunction. *Taxon* **50**: 1001-1018.

Taberlet P, Gielly L, Pautou G, Bouvet J. 1991. Universal primers for amplification of three non-coding regions of chloroplast DNA. *Plant Molecular Biology* **17**: 1105–1109.

White TJ, Bruns T, Lee S, Taylor J. 1990. Amplification and direct sequencing of fungal ribosomal RNA genes for phylogenetics. In: Innis MA, Gelfand DH, Sninsky JJ, White TJ, eds. *PCR Protocols*. Academic Press, San Diego, California, USA, pp. 315-322.
